# Association of GnRH agonists with depression and suicide/self-injury: a FAERS pharmacovigilance study

**DOI:** 10.1530/EC-25-0830

**Published:** 2026-03-18

**Authors:** Jinhua Liu, Liping Xue, Ruolin Chen, Ying Liu, Fanxiang Zeng, Peiguang Niu, Jintuo Zhou, Yanting Zhu, Jinhua Zhang, Huajiao Chen

**Affiliations:** ^1^ Department of Pharmacy, Fujian Maternity and Child Health Hospital, College of Clinical Medicine for Obstetrics & Gynecology and Pediatrics, Fujian Medical University, Fuzhou, China; ^2^ School of Pharmacy, Fujian Medical University, Fuzhou, China; ^3^ College of Letter and Science, University of California Davis, Davis, California, USA

**Keywords:** GnRH-a, depression, suicide, adverse event, FAERS, pharmacovigilance

## Abstract

**Background:**

The relationship between gonadotropin-releasing hormone agonists (GnRH-as) and depression and suicide/self-injury (DASSI) remains controversial. This study aimed to investigate this potential association using data from the FDA Adverse Event Reporting System (FAERS) database.

**Methods:**

Instances of DASSI linked with GnRH-as were identified from FAERS (2004Q1 to 2024Q1). Time-to-onset (TTO) analyses and disproportionality analysis (DPA) were employed to assess onset timing and signal values for adverse events. The influence of concurrent medications on DASSI was evaluated using the Ω shrinkage measure.

**Results:**

A total of 5,454 DASSI cases linked to GnRH-as were identified. TTO analyses showed earlier onset of DASSI in females, children (<18), and adults (18–65) compared to males and elders (>65) (females vs males: 3.5 (0.5–29.5) vs 120.5 (0.5–441.5) days, *P* < 0.001; children vs elders: 10.5 (0.5–78.5) vs 129.5 (0.5–490.5) days, *P* < 0.001; adults vs elders: 7.0 (0.5–81.0) vs 129.5 (0.5–490.5) days, *P* < 0.001). DPA revealed stronger signals for depression and suicide/self-injury in females and oncology patients compared to males and non-oncology patients. Co-medication analysis identified depressive interactions with multiple drug combinations.

**Conclusions:**

Although causality cannot be inferred from FAERS data and the results should be interpreted cautiously, we identified some signals of disproportionate reporting of DASSI for certain GnRH-a therapies. These hypothesis-generating findings may raise clinical awareness and inform a risk-informed monitoring framework (e.g., baseline assessment of mood and suicidality, patient/caregiver education on warning signs, and early follow-up) but require confirmation in well-designed epidemiologic studies.

## Introduction

Gonadotropin-releasing hormone agonists (GnRH-as) are synthetic, highly potent analogs of the natural hormone GnRH produced by the human hypothalamus. GnRH-as exhibit a high affinity for the GnRH receptor, initially inducing the release of luteinizing hormone (LH) and follicle-stimulating hormone (FSH). Continuous administration of GnRH-as leads to feedback inhibition, markedly reducing LH, FSH, and their associated effects, ultimately decreasing serum levels of androgens and estrogens. This makes GnRH-as effective in treating hormone-sensitive conditions, such as prostate cancer, endometriosis, uterine leiomyoma, central precocious puberty, and hormone receptor-positive breast cancer in premenopausal women (1, 2, 3). In addition to these established indications, GnRH-as have also been used in gender-affirming care, including pubertal suppression in adolescents with gender dysphoria and, in some settings, as part of androgen suppression in transgender women receiving estrogen therapy (4, 5). Despite their efficacy, the use of GnRH-as often accompanies adverse events (AEs), including hot flashes, decreased libido, headaches, fatigue, mood swings, erectile dysfunction, vaginal symptoms, and cardiovascular complications (6).

Sex hormone levels play a significant role in mental and behavioral processes, with fluctuations potentially increasing depression and suicidal thoughts or self-harm behaviors, which can be life-threatening (7, 8, 9). The suppression of the hypothalamic–pituitary–gonadal axis by GnRH-as and the resulting decrease in sex hormone synthesis may be associated with an increased risk of depression and suicide/self-harm and is cautioned in some GnRH-a drug labels. However, studies on this association have been mixed and often focus on specific populations or indications. Some research suggested an increased risk of depression with GnRH-a use in men with prostate cancer (10, 11), while others found no significant association (12, 13). Similarly, studies on women showed mixed results regarding mood changes with GnRH-a therapy (14, 15). Given the widespread use of GnRH-as and the conflicting findings on their psychological effects, it is essential for public health and clinical safety to investigate the potential link between GnRH-as and depression or suicide/self-harm.

The FDA Adverse Event Reporting System (FAERS) provides valuable real-world data on AEs, aiding researchers in identifying associations between specific drugs and AEs (16, 17). The present study employed the FAERS database to identify and analyze instances of depression and suicide/self-harm associated with GnRH-as, with the aim of evaluating their safety and providing evidence-based recommendations for clinical applications.

## Methods

### Data sources

**Figure 1:**
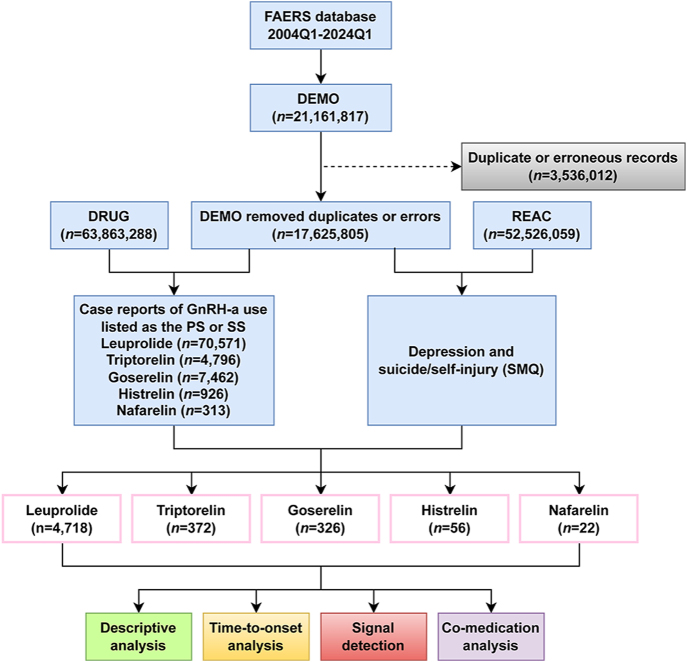
Data processing flow chart delineating the selection process of depression and suicide/self-injury post-GnRH-a treatment. DEMO, demographics; DRUG, drug; FAERS, the FDA Adverse Event Reporting System; GnRH-as, gonadotropin-releasing hormone agonists; PS, primary suspect; REAC, reaction; SMQs, standard MedDRA queries; and SS, secondary suspect.

This real-world, retrospective observational pharmacovigilance study used data from the FAERS database. FAERS is a publicly accessible database maintained by the US FDA, designed to collect and monitor reports of AEs related to drugs and other medical products. Its data usage is regulated by the FDA’s pharmacovigilance guidelines and policies. The FAERS database is updated quarterly and comprises seven datasets: demographic information, drug information, adverse drug reaction information, patient outcome information, report sources, drug therapy start and end dates, and indications for drug administration. All data are de-identified to protect patient privacy. As the data are publicly available and de-identified, no specific ethics approval is required for their use. Due to the nature of data updates, FAERS inevitably includes duplicate reports. The study analyzed data from 2004Q1 to 2024Q1, using R (version 4.4.0) to process 21,161,817 records. The data cleaning process followed the FDA-recommended method for removing duplicate reports. Relevant fields from the DEMO table, including PRIMARYID, CASEID, and FDA_DT, were selected. The records were sorted by CASEID, FDA_DT, and PRIMARYID. For reports with the same CASEID, the most recent FDA_DT was retained. When both CASEID and FDA_DT were identical, the report with the highest PRIMARYID was kept. After deduplication, 3,536,012 reports were removed, leaving a total of 17,625,805 reports for further analysis (Fig. 1).

### Screening of drugs and cases

Drug files were identified using both generic and trade names, focusing on FDA-approved GnRH-as (18, 19): leuprolide, triptorelin, goserelin, buserelin, histrelin, and nafarelin. In the FAERS database, AEs are standardized using preferred terms (PTs) from the Medical Dictionary for Regulatory Activities (MedDRA). Standard MedDRA queries (SMQs) group PTs to represent specific conditions. The study identified depression and suicide/self-injury (DASSI) as the SMQ of interest, with two subordinate SMQs: depression (excluding suicide/self-injury) (DE) and suicide/self-injury (SSI). The PTs included in the SMQs are shown in Supplementary Table S1 (see section on Supplementary materials given at the end of the article). The inclusion criteria for this study were as follows: i) GnRH-a was listed as either a primary or secondary suspect drug in the patient’s medication list and ii) DASSI occurred following GnRH-a administration. Only cases meeting both criteria were included in the study.

### Time-to-onset and cumulative distribution curve analysis

The time to onset (TTO) was defined as the interval between GnRH-a treatment start and DASSI occurrence, calculated using the formula ‘EVENT_DT − START_DT + 0.5’ (20, 21). We conducted goodness-of-fit tests on the collected latency data using Weibull, normal, gamma, and exponential distribution models to identify the most suitable model for TTO analysis. Parameters α (scale) and β (shape) characterized each distribution (22). When *β* < 1 and the upper limit of its 95% confidence interval (CI) < 1, the frequency of AEs increases initially but decreases over time, indicating an early failure type. Conversely, if *β* > 1 and the lower limit of its 95% CI > 1, the frequency of AEs progressively increases over time, suggesting a wear-out failure type. If β equals 1 or is near 1 and the 95% CI includes 1, it suggests a constant frequency of AEs throughout the treatment period with GnRH-as, indicating a random failure type.

Cumulative distribution curves were employed to illustrate TTO characteristics of DASSI following GnRH-a treatment across various sex and age groups. Furthermore, we compared the differences in DASSI onset times between individuals treated with GnRH-as and those not receiving such treatment.

### Disproportionality analysis

Disproportionality analysis (DPA) identified potential positive signals by comparing GnRH-as with other medications in FAERS. The frequency-dependent reporting odds ratio (ROR), proportional reporting ratio (PRR), and Bayesian confidence propagation neural network of information components (ICs) were used to assess the imbalance of specific AEs between GnRH-a cases and non-GnRH-a cases (23, 24). Detailed formulas and criteria for these algorithms are provided in Supplementary Table S2.

We performed sensitivity analyses to account for potential confounders. Given that most drugs caused gastrointestinal AEs, we excluded these cases to reduce their impact and avoid competitive bias (25). Furthermore, the primary indications for the included cases were prostate cancer, endometriosis, uterine leiomyoma, precocious puberty, and breast cancer. They are potential risk factors for DE and SSI. Several studies have reduced bias by limiting the indications (26, 27). Consequently, we restricted the dataset to patients with these five indications. Subgroup analyses based on gender and age further examined the influence of these factors on DE or SSI reporting.

### Co-medication analysis

In the two cohorts of DE or SSI cases associated with GnRH-as, we analyzed drug interactions between GnRH-as and the 20 most frequently used drugs, using the IC to assess the signal of DE or SSI for each drug. The Ω shrinkage measure was selected for drug–drug interaction (DDI) monitoring due to its consistent conservativeness demonstrated across multiple studies (28, 29). The procedure for calculating Ω is detailed in Supplementary Tables S3 and S4.

### Statistical analysis

In the TTO analysis, the Mann–Whitney U test and the Kruskal–Wallis test were employed to compare the median time to onset of DASSI between GnRH-a and non-GnRH-a patients, with stratifications based on sex and age cluster. The Benjamini–Hochberg procedure was applied to control the false-positive rate across the multiple comparisons. In DPA, a positive signal was identified when ROR_025_ > 1, *χ*^2^ > 4, and IC_025_ > 0 (30). In co-medication analysis, a positive signal was determined if IC_025_ > 0, with a significant interaction observed when Ω_025_ > 0. All analyses were conducted using R. A two-sided *P* value less than 0.05 was considered statistically significant.

## Results

### Descriptive analysis

We identified cases of DASSI associated with leuprolide (4,718), triptorelin (372), goserelin (326), histrelin (56), and nafarelin (22), while no cases were reported for buserelin. Triptorelin had the highest DASSI proportion (4.09%), followed by nafarelin (3.77%) and histrelin (3.25%), with goserelin having the lowest proportion (1.84%) (Supplementary Table S5). Despite the missing or unknown of some patient information, a slightly higher proportion of cases were reported in females compared to males (49.33 vs 46.09%; Table 1). Among the reports with available data, most cases were in the age group of 18–65 years (30.38%). The distribution of reported years shows a general upward trend, with 49.00% recorded between 2019 and 2024Q1. Non-healthcare professionals accounted for most of the reports (73.06%). Among GnRH-a-related DASSI cases, the most frequent indications were prostate cancer (39.99%) and endometriosis (24.50%). Lower proportions were observed for uterine leiomyoma (4.75%), precocious puberty (3.77%), and breast cancer (3.55%). Only a small fraction of cases involved gender dysphoria (0.38%) or anti-androgen therapy in transgender women (0.24%). Indications that were unknown or missing accounted for 19.53%. AE outcomes included hospitalization (15.36%), disability (4.51%), and death (3.59%), with goserelin showing the highest rates of hospitalization (29.45%), disability (18.71%), and death (6.44%).

**Table 1:** Case characterization of DASSI related to GnRH-as in the FAERS database.

Categories	Leuprolide	Triptorelin	Goserelin	Histrelin	Nafarelin	Total
	*n* (%)	*n* (%)	*n* (%)	*n* (%)	*n* (%)	*n* (%)
Number of reports	4,718	372	326	56	22	5,494
Sex						
Male	2,299 (48.73)	87 (23.39)	132 (40.49)	14 (25.00)	0 (0.00)	2,532 (46.09)
Female	2,239 (47.46)	278 (74.73)	131 (40.18)	40 (71.43)	22 (100.00)	2,710 (49.33)
Unknown or missing	180 (3.82)	7 (1.88)	63 (19.33)	2 (3.57)	0 (0.00)	252 (4.59)
Age category, years						
Juveniles < 18	231 (4.90)	91 (24.46)	0 (0.00)	23 (41.07)	0 (0.00)	345 (6.28)
Adults 18–65	1,461 (30.97)	40 (10.75)	150 (46.01)	0 (0.00)	18 (81.82)	1,669 (30.38)
Seniors > 65	1,124 (23.82)	21 (5.64)	78 (23.93)	4 (7.14)	0 (0.00)	1,227 (22.33)
Unknown or missing	1,902 (40.31)	220 (59.14)	98 (30.06)	29 (51.79)	4 (18.18)	2,253 (41.01)
Median (IQR)	51 (32–74)	9 (8–44)	53 (43–72)	10 (9–12)	39 (33–41)	/
Report year						
2004–2008	104 (2.20)	8 (2.15)	39 (11.96)	1 (1.79)	0 (0.00)	152 (2.77)
2009–2013	808 (17.13)	25 (6.72)	39 (11.96)	25 (44.64)	3 (13.64)	900 (16.38)
2014–2018	1,587 (33.64)	20 (5.38)	111 (34.05)	22 (39.29)	10 (45.45)	1,750 (31.85)
2019–2024Q1	2,219 (47.03)	319 (85.75)	137 (42.02)	8 (14.29)	9 (40.91)	2,692 (49.00)
Reporter*						
Healthcare professional	1,047 (22.19)	80 (21.51)	156 (47.85)	23 (41.07)	8 (36.36)	1,314 (23.92)
Non-healthcare professional	3,551 (75.26)	290 (77.96)	133 (40.80)	26 (46.43)	14 (63.64)	4,014 (73.06)
Unknown or missing	120 (2.54)	2 (0.54)	37 (11.35)	7 (12.50)	0 (0.00)	166 (3.02)
Indication						
Prostate cancer	2,056 (43.58)	35 (9.41)	100 (30.67)	6 (10.71)	0 (0.00)	2,197 (39.99)
Endometriosis	1,310 (27.77)	6 (1.61)	23 (7.06)	0 (0.00)	7 (31.82)	1,346 (24.50)
Uterine leiomyoma	241 (5.11)	0 (0.00)	19 (5.83)	0 (0.00)	1 (4.55)	261 (4.75)
Precocious puberty	154 (3.26)	21 (5.65)	0 (0.00)	32 (57.14)	0 (0.00)	207 (3.77)
Breast cancer	76 (1.61)	32 (8.60)	87 (26.69)	0 (0.00)	0 (0.00)	195 (3.55)
Off-label use	51 (1.08)	8 (2.15)	10 (3.07)	0 (0.00)	2 (9.09)	71 (1.29)
AUB/HMB and menstrual symptoms	60 (1.27)	0 (0.00)	4 (1.23)	0 (0.00)	5 (22.73)	69 (1.26)
Infertility and ART	31 (0.66)	5 (1.34)	0 (0.00)	0 (0.00)	5 (22.73)	41 (0.75)
Gender dysphoria	16 (0.34)	3 (0.81)	0 (0.00)	2 (3.57)	0 (0.00)	21 (0.38)
Anti-androgen therapy in transgender women	8 (0.17)	5 (1.34)	0 (0.00)	0 (0.00)	0 (0.00)	13 (0.24)
Unknown or missing	715 (15.15)	257 (69.09)	83 (25.46)	16 (28.57)	2 (9.09)	1,073 (19.53)
Outcomes^†^						
Death	169 (3.58)	7 (1.88)	21 (6.44)	0 (0.00)	0 (0.00)	197 (3.59)
Life-threatening	80 (1.70)	11 (2.96)	15 (4.60)	1 (1.79)	7 (31.82)	114 (2.07)
Hospitalization	682 (14.46)	62 (16.67)	96 (29.45)	3 (5.36)	1 (4.55)	844 (15.36)
Disability	179 (3.79)	8 (2.15)	61 (18.71)	0 (0.00)	0 (0.00)	248 (4.51)
Required intervention	46 (0.97)	0 (0.00)	2 (0.61)	0 (0.00)	0 (0.00)	48 (0.87)
Other serious illness	1,476 (31.28)	117 (31.45)	222 (68.10)	10 (17.86)	10 (45.45)	1,835 (33.40)
Not specified	2,749 (58.27)	203 (54.57)	41 (12.58)	42 (75.00)	5 (22.73)	3,040 (55.33)

* Healthcare professionals including physicians, pharmacists, and other healthcare professionals; non-healthcare professional including customers and lawyers.

^†^ Because a case may have one or more clinical outcomes, the percentage for this item may exceed 100%.

Abbreviations: ART, assisted reproductive technology; AUB, abnormal uterine bleeding; and HMB, heavy menstrual bleeding.

### Time-to-onset analysis

A total of 2,617 DASSI cases related to GnRH-a treatment was included in the TTO analysis. The results of the TTO analysis of DASSI for various GnRH-as, based on parameter distributions, are presented in Table 2. Data for nafarelin were limited (median (IQR) TTO = 0.5 (0.5–1.25) days; *n* = 6). Among the other four GnRH-as, goserelin showed the earliest median TTO with the narrowest range (median (IQR) TTO = 21.5 (1.5–101.5) days; *n* = 93), while triptorelin had the latest onset and the broadest range (median (IQR) TTO = 53.5 (1.0–407.0) days; *n* = 79). Except for nafarelin, the results of the goodness-of-fit performance tests (Supplementary Table S6) indicated that the Weibull model effectively described the DASSI latencies for all GnRH-as, which were categorized as an early failure type (Table 2). In contrast, nafarelin’s latencies fit the exponential distribution model, classifying them as random failure types.

**Table 2:** Time-to-onset analysis for different GnRH-as.

Drug				Best model				Failure type
		TTO (days)		Scale parameter		Shape parameter		
	Cases (*n*)	Median (IQR)	Min–max	*α*	95% CI	*β*	95% CI	
Leuprolide	2,424	31.5 (0.5–325.75)	0.5–6,008.5	85.54	75.96–95.13	0.38	0.36–0.39	Early failure
Triptorelin	79	53.5 (1.0–407.0)	0.5–2,161.5	114.39	49.53–179.26	0.41	0.34–0.48	Early failure
Goserelin	93	21.5 (1.5–101.5)	0.5–1,857.5	54.91	26.01–83.82	0.41	0.35–0.47	Early failure
Histrelin	15	42.5 (0.5–122.0)	0.5–1,277.5	65.68	−13.18–144.54	0.44	0.27–0.62	Early failure
Nafarelin	6	0.5 (0.5–1.25)	0.5–5.5	1.50	0.83–2.17	1.00	/	Random failure

Abbreviations: CI, confidence interval; IQR, interquartile range; *n*, number of reports with available time to onset; TTO, time to onset; *α*, scale parameter; and *β*, shape parameter.

When *β* < 1 and the upper limit of its 95% CI < 1, the frequency of AEs increases initially but decreases over time, indicating an early failure type. Conversely, if *β* > 1 and the lower limit of its 95% CI > 1, the frequency of AEs progressively increases over time, suggesting a wear-out failure type. If *β* ≈ 1 or the 95% CI includes 1, it suggests a constant frequency of AEs throughout the treatment period, indicating a random failure type.

**Figure 2:**
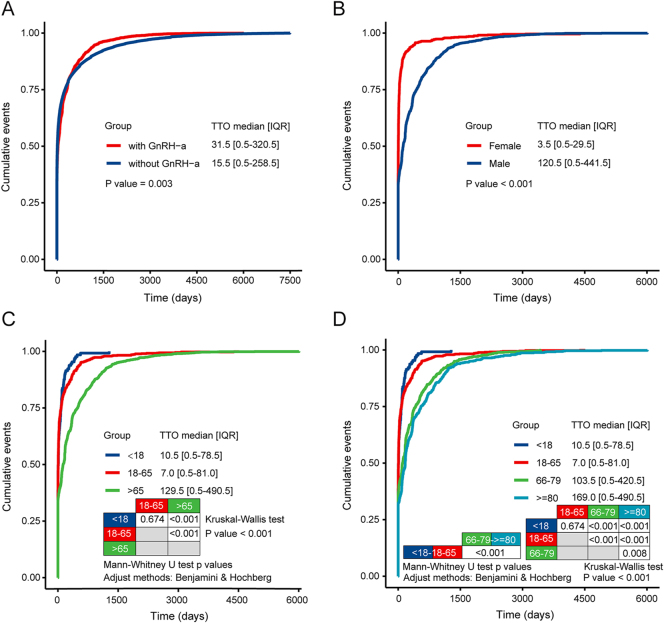
Time-to-onset analysis of DASSI. Cumulative distribution curves of DASSI onset: (A) GnRH-a vs non-GnRH-a therapy. (B) By sex group among GnRH-a recipients. (C and D) By age group among GnRH-a recipients. In (D), the age group of >65 years is subdivided into two categories: ‘66–79 years old’ and ‘>= 80 years old’. Two-group comparisons used the Mann–Whitney U test. For analyses with >2 groups, an overall Kruskal–Wallis test was performed, followed by pairwise comparisons with Benjamini–Hochberg correction for multiple testing. DASSI, depression and suicide/self-injury; FAERS, FDA Adverse Event Reporting System; GnRH-as, gonadotropin-releasing hormone agonists; and TTO, time to onset.

The cumulative distribution curves of DASSI showed that the median TTO was longer in GnRH-a-treated patients than in untreated ones (median (IQR) TTO = 31.5 (0.5–320.5) vs 15.5 (0.5–258.5) days; *P* = 0.003; Fig. 2A). Stratified analysis by sex and age revealed that the median DASSI onset was earlier in females compared to males (median (IQR) TTO = 3.5 (0.5–29.5) vs 120.5 (0.5–441.5) days; *P* < 0.001; Fig. 2B). Age also significantly influenced TTO (Fig. 2C and D), with elderly patients (over 65 years) experiencing later DASSI onset (median (IQR) TTO = 129.5 (0.5–490.5) days) compared to those aged 18–65 years (median (IQR) TTO = 7.0 (0.5–81.0) days; *P* < 0.001) or under 18 years (median (IQR) TTO = 10.5 (0.5–78.5) days; *P* < 0.001).

### Disproportionality analysis

We investigated the association between GnRH-as and DE or SSI. A preliminary analysis was conducted to compare GnRH-as with other medications in the FAERS database. Leuprolide (ROR_025_ = 1.18; *χ*^2^ = 206.80; IC_025_ = 0.23) and triptorelin (ROR_025_ = 1.46; *χ*^2^ = 100.92; IC_025_ = 0.51) displayed disproportionate reporting signals for DE, while goserelin, histrelin, and nafarelin did not. In contrast, none of the five GnRH-as showed signals for SSI (Table 3).

**Table 3:** Primary analysis of the relationship between GnRH-as and DASSI.

Drug	a	b	c	d	ROR	ROR_025_	PRR	*χ* ^2^	IC	IC_025_	IC_975_
Depression											
Leuprolide	5,671	201,481	1,185,681	51,133,226	1.21	**1.18**	1.21	**206.80**	0.27	**0.23**	0.30
Triptorelin	456	12,224	1,190,896	51,322,483	1.61	**1.46**	1.59	**100.92**	0.66	**0.51**	0.78
Goserelin	381	23,908	1,190,971	51,310,799	0.69	0.62	0.69	53.64	−0.53	−0.70	−0.41
Histrelin	67	2,176	1,191,285	51,332,531	1.33	1.04	1.32	5.23	0.39	−0.01	0.69
Nafarelin	32	922	1,191,320	51,333,785	1.50	1.05	1.48	5.08	0.55	−0.04	0.97
Suicide/self-injury											
Leuprolide	397	206,755	296,941	52,021,966	0.34	0.30	0.34	518.01	−1.56	−1.73	−1.44
Triptorelin	63	12,617	297,275	52,216,104	0.88	0.68	0.88	1.08	−0.19	−0.60	0.11
Goserelin	67	24,222	297,271	52,204,499	0.49	0.38	0.49	36.37	−1.03	−1.44	−0.74
Histrelin	6	2,237	297,332	52,226,484	0.47	0.21	0.47	3.55	−1.02	−2.44	−0.11
Nafarelin	4	950	297,334	52,227,771	0.74	0.28	0.74	0.37	−0.39	−2.16	0.69

Abbreviations: *IC*, information component; *IC_025_*, lower side of the 95% confidence interval for IC; *IC_975_*, upper side of the 95% confidence interval for IC; *PRR*, proportional reporting ratio; *ROR*, reporting odds ratio; ROR_025_, lower side of the 95% confidence interval for ROR; and *χ*^2^, chi-squared. Bold values denote statistics for which all three prespecified criteria for statistical significance were met: ROR_025_ > 1, IC_025_ > 0, and *χ*^2^ > 4.

**Figure 3:**
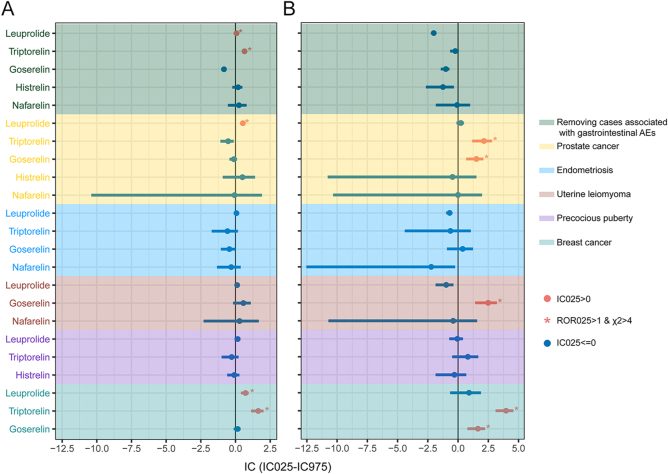
IC_025_ for GnRH-as in the sensitivity analyses. (A) IC_025_ in DE. (B) IC_025_ in SSI. When ROR_025_ > 1, *χ*^2^ > 4, and IC_025_ > 0, a disproportionate reporting signal was detected. DE, depression; FAERS, FDA Adverse Event Reporting System; GnRH-as, gonadotropin-releasing hormone agonists; IC, information component; IC_025_, lower side of the 95% CI for IC; ROR, reporting odds ratio; ROR_025_, lower side of the 95% CI for ROR; SSI, suicide/self-injury; and *χ^2^*, chi-squared.

In sensitivity analyses (Fig. 3A and B), results were consistent with preliminary findings after excluding gastrointestinal AEs. Unexpected results arose when restricted by indication: no association was found between GnRH-as and DE or SSI in patients with endometriosis or precocious puberty. However, in prostate cancer patients, leuprolide was associated with DE (ROR_025_ = 1.68; *χ*^2^ = 441.03; IC_025_ = 0.46), while triptorelin (ROR_025_ = 3.01; *χ*^2^ = 40.61; IC_025_ = 1.17) and goserelin (ROR_025_ = 1.91; *χ*^2^ = 22.22; IC_025_ = 0.67) were associated with SSI. SSI signals were identified in patients with uterine leiomyoma treated with goserelin (ROR_025_ = 4.44; *χ*^2^ = 55.78; IC_025_ = 1.41). Among breast cancer patients, leuprolide was associated with DE (ROR_025_ = 1.37; *χ*^2^ = 26.46; IC_025_ = 0.39), while triptorelin showed disproportionate reporting signals of DE (ROR_025_ = 2.42; *χ*^2^ = 61.67; IC_025_ = 1.12) and SSI (ROR_025_ = 19.02; *χ*^2^ = 429.37; IC_025_ = 3.10). Goserelin was also associated with SSI (ROR_025_ = 2.05; *χ*^2^ = 24.98; IC_025_ = 0.77). Detailed sensitivity analysis calculations are provided in Supplementary Tables S7 and S8.

**Figure 4:**
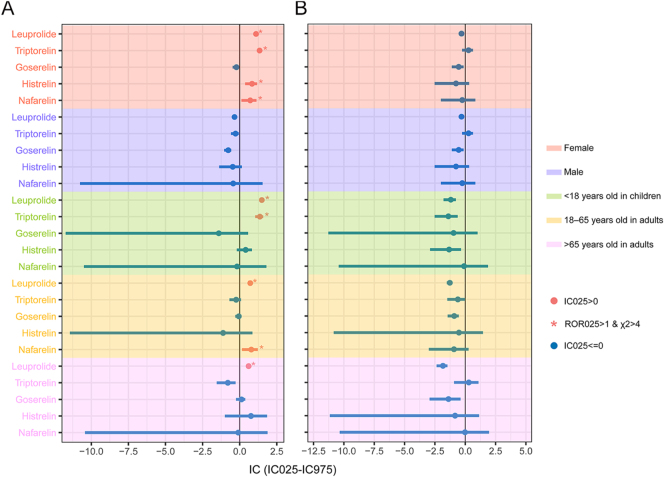
Subgroup analyses of IC_025_ for GnRH-as. (A) IC_025_ in DE. (B) IC_025_ in SSI. When ROR_025_ > 1, *χ*^2^ > 4, and IC_025_ > 0, a disproportionate reporting signal was detected. DE, depression; FAERS, FDA Adverse Event Reporting System; GnRH-as, gonadotropin-releasing hormone agonists; IC, information component; IC_025_, lower side of the 95% CI for IC; ROR, reporting odds ratio; ROR_025_, lower side of the 95% CI for ROR; SSI, suicide/self-injury; and *χ*^2^, chi-squared.

Subgroup analyses were conducted based on age and sex. In females, four GnRH-as exhibited signals of DE: leuprolide (ROR_025_ = 2.13; *χ*^2^ = 1,788.83; IC_025_ = 1.04), triptorelin (ROR_025_ = 2.35; *χ*^2^ = 319.50; IC_025_ = 1.16), histrelin (ROR_025_ = 1.38; *χ*^2^ = 18.43; IC_025_ = 0.37), and nafarelin (ROR_025_ = 1.18; *χ*^2^ = 8.48; IC_025_ = 0.12). No such signals were detected in males. Leuprolide was associated with DE across all age groups (<18 years: ROR_025_ = 2.64; *χ*^2^ = 420.05; IC_025_ = 1.31; 18–65 years: ROR_025_ = 1.59; *χ*^2^ = 474.59; IC_025_ = 0.64; >65 years: ROR_025_ = 1.44; *χ*^2^ = 218.23; IC_025_ = 0.50). Triptorelin was linked to DE in children (ROR_025_ = 2.21; *χ*^2^ = 108.87; IC_025_ = 1.04), and nafarelin indicated a depressive signal in adults aged 18–65 years (ROR_025_ = 1.22; *χ*^2^ = 9.33; IC_025_ = 0.16). No association between GnRH-as and SSI was observed across any age or sex groups (Fig. 4A and B). Detailed subgroup analysis calculations are provided in Supplementary Tables S9 and S10.

### Co-medication analysis

**Figure 5:**
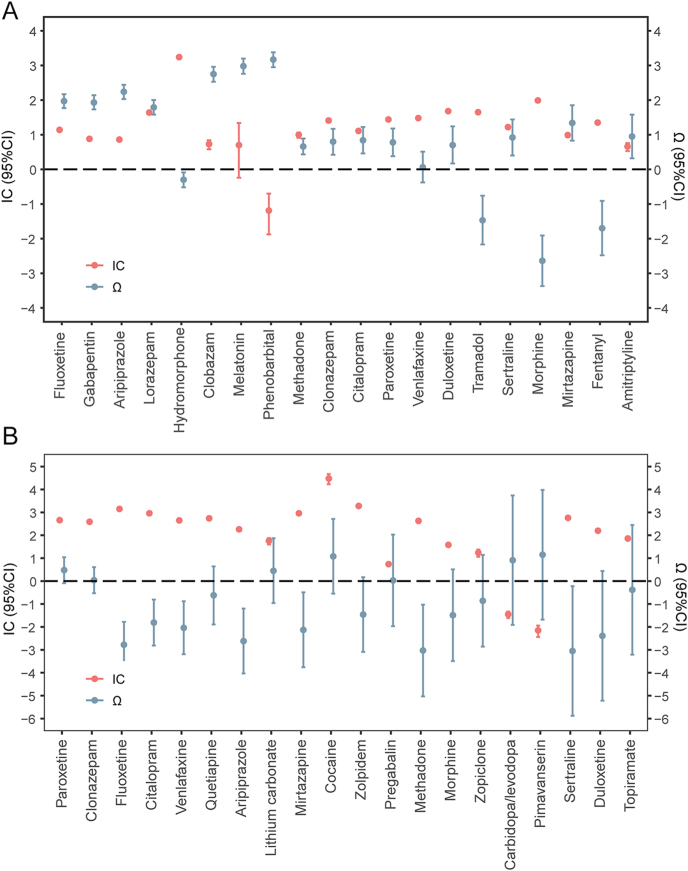
Forest plot of ICs and Ωs for neurological medications combined with GnRH-as. (A) In patients with DE. (B) In patients with SSI. When IC_025_ > 0, a disproportionate reporting signal was detected. When Ω_025_ > 0, a drug–drug interaction signal was considered significant. DE, depression; GnRH-as, gonadotropin-releasing hormone agonists; IC, information component; IC_025_, lower side of the 95% CI for IC; SSI*,* suicide/self-injury; and Ω_025_, lower side of the 95% CI for Ω.

We conducted a co-medication analysis of all GnRH-a-related reports concerning DE or SSI. Neurologic medications, including fluoxetine, aripiprazole, and clonazepam, were identified as commonly used. Therefore, our co-medication analysis focused on the 20 neurologic medications most frequently used. Among patients with DE on GnRH-as, 251 had a history of neurologic medication use. Detailed information on sex, age, concomitant medications, and indications for GnRH-as in these patients is shown in Supplementary Fig. S1. Subsequently, we identified depressive interactions between GnRH-as and 15 neurologic drugs using Ω shrinkage (Fig. 5A). The IC and Ω values for these neurologic drugs are detailed in Supplementary Table S11. The interactions involved fluoxetine, gabapentin, aripiprazole, lorazepam, clobazam, melatonin, phenobarbital, methadone, clonazepam, citalopram, paroxetine, duloxetine, sertraline, mirtazapine, and amitriptyline. DE signals were observed for all the mentioned drugs, except for melatonin (IC_025_ = −0.24) and phenobarbital (IC_025_ = −1.88).

Of the patients who experienced SSI while on GnRH-as, 59 were also taking neurologic medications (Supplementary Fig. S2). Subsequent Ω shrinkage indicated no SSI interactions between GnRH-as and neurologic drugs (all Ω_025_ < 0; Fig. 5B). Among the 20 neurologic drugs, disproportionate signals of SSI were observed for most, with the exceptions of carbidopa/levodopa (IC_025_ = −1.61) and pimavanserin (IC_025_ = −2.44). The IC and Ω values for these drug combinations are given in Supplementary Table S12.

## Discussion

This is the first study to utilize FAERS, the world’s largest pharmacovigilance database, to explore the association between GnRH-a treatment and DASSI, encompassing both DE and SSI. Recognizing the relationship between GnRH-as and DASSI is crucial for clinical practice, as underestimating the association could jeopardize patients’ mental health, while overestimating it might unnecessarily limit treatment options.

Initially, our comparative analysis of clinical characteristics revealed a slightly higher proportion of DASSI reports from females than from males (49.33 vs 46.09%), aligning with epidemiological surveys on depression and suicide attempts (31, 32). The age group of 18–65 years was predominant, a finding consistent with the epidemiology of depression, as studies indicate that suicide and self-harm are more prevalent in this age range (33, 34). Furthermore, between 2004Q1 and 2024Q1, 83,754 cases involving GnRH-as were reported, with the proportions of females, males, and unknown being 27.17, 61.69, and 11.14%, respectively. The proportion of children, adults, elderly individuals, and those of unknown age was 3.62, 18.88, 29.91, and 47.59%, respectively. We believe that the higher reporting rates among females and adults may not be related to the distribution of reports. Nearly half of the reports were from the past five years, possibly due to increased GnRH-a use or the COVID-19 pandemic (35, 36, 37). Through analyzing the clinical characteristics of the patients involved, we obtained a preliminary understanding of the potential correlation between GnRH-as and DASSI.

Next, we examined the latency of DASSI using TTO analysis to help clinicians and patients understand its onset time for better prevention and monitoring. GnRH-a drugs have similar pharmacological mechanisms; they initially induce a transient increase in androgen and estrogen levels followed by a decrease with prolonged use; and these hormonal fluctuations may elevate the risk of DASSI (38, 39). Research has indicated that GnRH-a-associated DASSI might involve serotonin-mediated changes in mental status and affective cognition (11). If the mechanisms underlying GnRH-a-associated DASSI are consistent, the pathogenic models should be similar. However, our goodness-of-fit tests showed that all but nafarelin (classified as a random failure model) were early failure models. This indicates that the link between GnRH-a and DASSI is not attributable to a single mechanism. Further research is needed to clarify this association.

The subsequent cumulative distribution curve analysis revealed that DASSI associated with GnRH-as had a later median onset compared to other medications. This delay may be linked to the initial stimulatory effect of GnRH-as and subsequent hormone level decline. Moreover, long-acting depot formulations, such as leuprolide and triptorelin, along with subcutaneous implants such as histrelin, are frequently prescribed GnRH-a formulations for patients. The delayed-release characteristic of these long-acting agents may contribute to the delayed phenomenon. Although the precise mechanism underlying GnRH-a-associated DASSI remains unclear, the delayed onset underscores the imperative for continuous monitoring of patients’ mental health during GnRH-a treatment.

In addition, we identified gender- and age-specific characteristics of the onset timing of DASSI. Female participants exhibited earlier DASSI manifestations than male subjects treated with GnRH-as. Estrogen levels have been associated with mood swings (40), and estrogen-related affective changes may also depend on individual sensitivity to hormonal fluctuations. Given that females experience cyclic ovarian hormone variability and may be more vulnerable to mood symptoms during reproductive transitions, we hypothesized that heightened sensitivity to estrogen fluctuations may contribute to earlier DASSI onset in women. One study indicated that women might be particularly vulnerable to depression during reproductive events, such as premenstrual, postpartum, and menopausal transitions, partly due to heightened sensitivity to significant hormonal fluctuations (41). Mechanistic hormone manipulation studies further suggest that, in susceptible women, changes in ovarian steroid exposure (including withdrawal or add-back of estradiol/progesterone) can precipitate depressive symptoms, despite hormone levels being within physiologic ranges (42, 43, 44). In addition, a community-based longitudinal study reported that depressive symptoms increased during the menopausal transition and decreased after menopause, with hormone trajectory analyses suggesting that a changing hormonal milieu may contribute to dysphoric mood during the transition (45). Biologically, estrogen may influence affective regulation by modulating central serotonergic neurotransmission, including serotonin synthetic capacity via regulation of tryptophan hydroxylase-2 expression, and by interacting with neurotrophic and neuroplasticity pathways, such as brain-derived neurotrophic factor (46, 47, 48). Further mechanistic and prospective studies are warranted to clarify sex-specific vulnerability and to inform risk mitigation strategies that may reduce sex-based inequities. Separately, adolescent and adult populations also exhibited earlier DASSI onset compared to older patients, although age-related factors remain unclear. The TTO analysis supported the earlier onset of DASSI in female, adolescent, and adult patients, underscoring the need for vigilant monitoring and early detection in these groups.

We conducted an exploratory analysis of GnRH-as alongside all drugs in the FAERS database, employing the methodology from previous pharmacovigilance studies (30). To enhance result accuracy, we modified the outcome indicator in the DPA from DASSI to DE and SSI. The DPA findings indicated the presence of DE signals in both leuprolide and triptorelin. These findings suggested a potential association between GnRH-as and DE, noting potential variations in signals among different GnRH-a formulations. We conducted a series of sensitivity analyses to consider the masking effect of gastrointestinal events and the potential influence of prior diseases on the occurrence of DE or SSI. Unexpectedly, no DE or SSI signals were observed with any GnRH-as in non-tumor patients with endometriosis and precocious puberty. However, DE or SSI signals were detected for certain GnRH-as in tumor patients with prostate cancer, uterine smooth muscle tumors, and breast cancer. This indicated that indication might be a confounding factor, making oncology patients more susceptible to the effects of GnRH-as compared to non-oncology patients, thereby leading to a positive signal for DE or SSI. Previous research has indicated that patients with prostate cancer, uterine leiomyoma, and breast cancer were more susceptible to depressive symptoms and suicidal tendencies (49, 50, 51, 52). Psychological issues often arise after a cancer diagnosis, with fears of death, pain, side effects, and role changes potentially leading to depression or suicidal ideation (53), and using GnRH-as during such a vulnerable mental state may intensify the emotional burden. The observed differences in indications highlighted the need for rigorous monitoring of the mental health of GnRH-a recipients with tumors, who were more susceptible to DE or SSI. Subsequent subgroup analyses indicated that none of the GnRH-a recipients across all sex and age groups exhibited signaling with SSI. Conversely, nearly all GnRH-as in the female group showed disproportionate signaling for DE, in contrast to the male group, where all GnRH-as were negative. An association between partial GnRH-as and DE was identified across all age groups. The validity of the above analysis further confirmed that sex and age may be potential confounders for DE.

Finally, we analyzed the top 20 concomitant medications with the highest reported frequency of DE or SSI associated with GnRH-as. Our initial examination revealed a range of neurologic medications that exhibited clear signals for DE or SSI. Previous studies have indicated that individuals using neurologic medications may have an increased risk of depressive symptom or suicide/self-harm (54). Consequently, we focused on the 20 most used neurologic medications, finding 251 cases of DE and 59 cases of SSI related to GnRH-as. We further investigated potential interaction signals between GnRH-as and neurologic drugs using the Ω shrinkage measure. The results showed that 15 drug combinations in the DE group exhibited Ω_025_ > 0. Interestingly, among these combinations, melatonin and phenobarbital, while not having disproportionately reported signals for DE, surprisingly resulted in increased reports of DE when they were combined with GnRH-as. While we could not fully explain this result, the various drug combinations identified underscore the need for vigilant monitoring of patients’ mental status when concomitant use of GnRH-as and potentially harmful combinations is medically necessary. Conversely, in the SSI group, no potential interactions between GnRH-as and neurologic medications were observed, possibly due to limited data. Further research is needed to clarify these findings.

This study has several limitations. First, FAERS is a spontaneous reporting system and is therefore subject to underreporting, misclassification, incomplete records, and potential duplicate submissions, which may bias signal estimates (55, 56). Because FAERS is publicly accessible and de-identified, ethics approval is generally not required; however, limited clinical details and non-standardized reporting constrain interpretability. Second, our analyses are intended for signal detection and cannot establish causality or estimate incidence, and FAERS does not permit construction of an ideal indication-matched untreated control group; therefore, the absence of a true control cohort is a major limitation. Third, residual confounding is likely – particularly for oncology indications, in which baseline risks of depressive symptoms and suicidality are inherently higher – and key determinants (e.g., cancer stage/severity, treatment context, concomitant medications, prior psychiatric history, and symptom burden) are unavailable or incompletely captured. Fourth, although we applied the Benjamini–Hochberg correction for multiple pairwise comparisons in the time-to-onset analyses, residual false-positive findings may remain; thus, these results should be interpreted as hypothesis-generating. Finally, formulation-specific analyses were not feasible because FAERS does not reliably record formulation details. In addition, secular influences – including the COVID-19 pandemic – may have affected background rates of depression and suicidality, but such effects cannot be adequately controlled for in FAERS.

## Conclusion

In summary, although causality cannot be inferred from FAERS data, our analyses identified signals of disproportionate reporting of DASSI for certain GnRH-a therapies. TTO analyses indicated an earlier onset time of DASSI in women, children, and adults. DPA indicated heterogeneity in DE and SSI signal strength across GnRH-a types, age groups, sexes, and indications, with stronger signals observed in women and oncology patients. Co-medication analyses further identified drug combinations associated with higher reported DE. Although these findings are hypothesis-generating and require confirmation in well-designed epidemiologic studies, they may raise clinical awareness and inform a risk-informed approach to monitoring. Individualized attention to DASSI symptoms across different GnRH-a recipient groups may be helpful, potentially facilitating earlier recognition of concerning symptoms and timely supportive management. Potential approaches include baseline assessment of mood and suicidality, education of patients and caregivers about warning signs, and early follow-up. Closer monitoring could be considered for higher-signal strata (e.g., women or oncology indications), those with relevant psychiatric history, or those receiving concomitant medications that may affect mood.

## Supplementary materials



## Declaration of interest

The authors declare that there is no conflict of interest that could be perceived as prejudicing the impartiality of the work reported.

## Funding

This study was supported by Fujian Provincial Health Technology Project (No. 2022GGA034), Joint Funds for the Innovation of Science and Technology of Fujian Province (No. 2024Y9551), and Fujian Provincial Natural Science Foundation of China (No. 2025J011148).

## Author contribution statement

Jinhua Liu conceived the study, designed the methodology, performed the investigation, and wrote the original draft of the manuscript. Liping Xue, Ruolin Chen, and Ying Liu performed the investigation and validation and obtained the software. Fanxiang Zeng and Yanting Zhu curated the data, performed formal analysis, and carried out visualization. Peiguang Niu and Jintuo Zhou curated the data and performed formal analysis and validation. Jinhua Zhang designed the methodology, supervised the study, and wrote, reviewed, and edited the manuscript. Huajiao Chen conceived and supervised the study, acquired funding, and wrote, reviewed, and edited the manuscript.

## Data availability

The datasets generated and analyzed during this study are available from the corresponding author (H Chen) upon reasonable request.

## Ethics statement

Ethical approval was not required for this study, as the data analyzed were de-identified records from a publicly available database.
