# Supplementary material for: Association of GnRH agonists with depression and suicide/self-injury: a FAERS pharmacovigilance study
Source: Endocr Connect. 2026 Mar 18;15(3):e250830. doi: 10.1530/EC-25-0830 (PMC13011796; doi:10.1530/EC-25-0830)
Supplement: Supplementary file 1 [file supplementary_materials.pdf]

# Online Supplementary Information

**Article title:** Association of GnRH agonists with depression and suicide/self-injury: a FAERS pharmacovigilance study

**Journal name:** *Endocrine Connections*


- Table S1……………………………………………………………..……..…..Page 2
- Table S2…………………………………………………………..……..……Page 3
- Table S3…………………………………………………………..………..…Page 4
- Table S4………………………………………………………..…………..…Page 4
- Table S5..……………………………………………………….…..……..…..Page 4
- Table S6..……………………………………………………….…..…..……..Page 5
- Table S7 ………………………………………………………..…...….…….Page 6–7
- Table S8 ……………………………………………………..……………….Page 8–9
- Table S9 …………………………………………………..…………………Page 10–11
- Table S10 ……………………………………………….……..…………….Page 12–13
- Fig. S1 ………………………..…………………..…………….……..………Page 14
- Table S11 ……………………………………………….……..…………….Page 15
- Fig. S2 ……………………..……..…………………..…………………...……Page 16
- Table S12 ……………………………………………….……..…………….Page 17

**Table S1** List of all adverse drug reactions of DASSI included in the study

| Upper SMQ | Lower SMQ | PT |
|---|---|---|
| Depression and suicide/self-injury | Depression (excl suicide and self-injury) | Activation syndrome; Adjustment disorder with depressed mood; Adjustment disorder with mixed anxiety and depressed mood; Agitated depression; Anhedonia; Antidepressant therapy; Childhood depression; Decreased interest; Depressed mood; Depression; Depression postoperative; Depression rating scale score increased; Depressive symptom; Discouragement; Dysphoria; Electroconvulsive therapy; Feeling guilty; Feeling of despair; Feelings of worthlessness; Helplessness; Major depression; Menopausal depression; Mixed anxiety and depressive disorder; Organic depression; Perinatal depression; Persistent depressive disorder; Post stroke depression; Postictal depression; Transcranial direct current stimulation; Affect lability; Alcohol abuse; Alcohol poisoning; Alcohol problem; Alcohol rehabilitation; Alcohol use disorder; Alcoholic coma; Alcoholism; Apathy; Blunted affect; Chronic idiopathic pain syndrome; Constricted affect; Crying; Disturbance in attention; Drug abuse; Drug abuser; Drug dependence; Drug dependence, antepartum; Drug dependence, postpartum; Drug use disorder, postpartum; Drug use disorder, antepartum; Drug use disorder; Dysania; Dyssomnia; Emotional distress; Emotional poverty; Hypersomnia; Hyposomnia; Increased need for sleep; Initial insomnia; Intentional product misuse; Intentional product use issue; Listless; Maternal use of illicit drugs; Memory impairment; Middle insomnia; Mood altered; Mood swings; Morose; Negative thoughts; Neglect of personal appearance; Paradoxical insomnia; Poor quality sleep; Psychiatric care; Psychomotor hyperactivity; Psychomotor retardation; Psychosocial support; Psychotherapy; Self esteem decreased; Self-destructive behaviour; Sense of a foreshortened future; Separation anxiety disorder; Substance abuse; Substance abuser; Substance dependence; Substance use; Substance use disorder; Substance-induced mood disorder; Tearfulness; Terminal insomnia |
| | Suicide/self-injury | Assisted suicide; Columbia suicide severity rating scale abnormal; Completed suicide; Depression suicidal; Intentional overdose; Intentional self-injury; Poisoning deliberate; Self-injurious ideation; Suicidal behaviour; Suicidal ideation; Suicide attempt; Suicide threat; Suspected suicide; Suspected suicide attempt |

Abbreviations: *DASSI*, depression and suicide/self-injury. *PT*, preferred terms; *SMQ*, Standard MedDRA Queries

**Table S2** Detailed formulas of disproportionality method

| Algorithms | Formula | Criteria |
|---|---|---|
| ROR | $ROR = \frac{ad}{bc} \#(1)$<br>$SE(lnROR) = \sqrt{\left(\frac{1}{a}+\frac{1}{b}+\frac{1}{c}+\frac{1}{d}\right)} \#(2)$<br>$ROR_{025} = e^{lnROR-1.96SE(lnROR)} \#(3)$<br>$ROR_{975} = e^{lnROR+1.96SE(lnROR)} \#(4)$ | $ROR_{025} > 1$, $\chi^2 > 4$, and $IC_{025} > 0$ |
| PRR | $PRR = \frac{a(c+d)}{c(a+b)} \#(5)$<br>$\chi^2 = \frac{(ad-bc)^2(a+b+c+d)}{(a+b)(a+c)(b+d)(c+d)} \#(6)$ | |
| BCPNN | $IC = log_2 \frac{a+0.5}{N_{expected}+0.5} \#(7)$<br>$IC_{025} = IC - 3.3 * (a+0.5)^{-1/2} - 2 * (a+0.5)^{-3/2} \#(8)$<br>$IC_{975} = IC + 2.4 * (a+0.5)^{-1/2} - 0.5 * (a+0.5)^{-3/2} \#(9)$ | |

Note: a, number of reports involving target drugs and target adverse drug reactions; b, number of reports involving target drugs and other adverse drug reactions; c, number of reports involving target adverse drug reactions and other drugs; d, number of reports involving other drugs and other adverse drug reactions

Abbreviations: *BCPNN*, Bayesian confidence propagation neural network; *IC*, information component; $IC_{025}$, lower side of the 95% confidence interval for IC; $IC_{975}$, upper side of the 95% confidence interval for IC; $N_{expected}$, expected number of reports involving target drugs and target adverse drug reactions; *PRR*, Proportional Reporting Ratio; *ROR*, Reporting Odds Ratio; $ROR_{025}$, lower side of the 95% confidence interval for ROR; $ROR_{975}$, upper side of the 95% confidence interval for ROR; $\chi^2$, chi-squared

**Table S3** The 4x2 contingency table for assessing drug-drug interactions signaling assays

| | Target AEs | Other AEs | Total |
|---|---|---|---|
| **Drug A and Drug B** | $n_{111}$ | $n_{110}$ | $n_{11+}$ |
| **Only Drug A** | $n_{101}$ | $n_{100}$ | $n_{10+}$ |
| **Only Drug B** | $n_{011}$ | $n_{010}$ | $n_{01+}$ |
| **Neither Drug A nor Drug B** | $n_{001}$ | $n_{000}$ | $n_{00+}$ |
| **Total** | $n_{++1}$ | $n_{++0}$ | $n_{+++}$ |

Note: n, number of reports

Abbreviations: *AEs*, adverse events

**Table S4** Detailed formulas of Ω shrinkage measure

| Algorithms | Formula | Criteria |
|---|---|---|
| Ω | $\Omega = log_2 \frac{n_{111} + 0.5}{E_{111} + 0.5} \#(10)$ <br> $f_{00} = \frac{n_{001}}{n_{00+}},\ f_{10} = \frac{n_{101}}{n_{10+}},\ f_{01} = \frac{n_{011}}{n_{01+}},\ f_{11} = \frac{n_{111}}{n_{11+}}\ \#(11)$ <br> $g_{11} = 1 - \frac{1}{max\left(\frac{f_{00}}{1-f_{00}}, \frac{f_{10}}{1-f_{10}}\right) + max\left(\frac{f_{00}}{1-f_{00}}, \frac{f_{01}}{1-f_{01}}\right) - \frac{f_{00}}{1-f_{00}} + 1} \#(12)$ <br> $E_{111} = g_{11} \times n_{11+} \#(13)$ <br> $\Omega_{025} = \Omega - \frac{\phi(0.975)}{\ln(2)\sqrt{n_{111}}} \#(14)$ | $\Omega_{025} > 0$ |

Abbreviations: $\Omega_{025}$, lower side of the 95% confidence interval for Ω

**Table S5** Proportion of target AEs to total AEs for each GnRH-a

| Drug | Target AEs | Total AEs | Proportion (%) |
|---|---|---|---|
| **Leuprolide** | 6068 | 207152 | 2.93 |
| **Triptorelin** | 519 | 12680 | 4.09 |
| **Goserelin** | 448 | 24289 | 1.84 |
| **Histrelin** | 73 | 2243 | 3.25 |
| **Nafarelin** | 36 | 954 | 3.77 |

Abbreviations: *AEs*, adverse events; *GnRH-a*, gonadotropin-releasing hormone agonists

**Table S6** Performance tests for goodness-of-fit of four parametric distribution models

| Model name | AICc | BIC | -2*Log L | Selection evidence |
|---|---|---|---|---|
| **For depression and suicide with leuprolide** | | | | |
| Weibull | 22757.12 | 22768.69 | 22753.11 | Weibull model presented the smallest AICs, showed a good fit |
| Normal | 33724.75 | 33736.33 | 33720.74 | |
| Gamma | 22812.46 | 22824.03 | 22808.45 | |
| Exponent | 28345.64 | 28351.43 | 28343.64 | |
| **For depression and suicide with triptorelin** | | | | |
| Weibull | 807.70 | 812.28 | 803.54 | Weibull model presented the smallest AICs, showed a good fit |
| Normal | 1090.38 | 1094.96 | 1086.22 | |
| Gamma | 809.63 | 814.21 | 805.47 | |
| Exponent | 945.94 | 948.26 | 943.89 | |
| **For depression and suicide with goserelin** | | | | |
| Weibull | 906.53 | 911.47 | 902.40 | Weibull model presented the smallest AICs, showed a good fit |
| Normal | 1306.83 | 1311.76 | 1302.70 | |
| Gamma | 917.42 | 922.35 | 913.29 | |
| Exponent | 1095.85 | 1098.35 | 1093.81 | |
| **For depression and suicide with histrelin** | | | | |
| Weibull | 163.16 | 163.57 | 158.16 | Weibull model presented the smallest AICs, showed a good fit |
| Normal | 219.98 | 220.40 | 214.98 | |
| Gamma | 163.60 | 164.01 | 158.60 | |
| Exponent | 183.65 | 184.04 | 181.34 | |
| **For depression and suicide with nafarelin** | | | | |
| Weibull | 24.85 | 20.44 | 16.85 | Exponent model presented the smallest AICs, showed a good fit |
| Normal | 32.25 | 27.83 | 24.25 | |
| Gamma | 24.83 | 20.41 | 16.83 | |
| Exponent | 22.87 | 18.66 | 16.87 | |

Note: AICc, Corrected Akaike Information Criterion; BIC, Bayesian Information Criterion; L, Log-likelihood. All three of these serve as indicators for the goodness-of-fit test, with a smaller AICc indicating a better model fit

**Table S7** Raw data and calculations for sensitivity analyses of DE

| Drug | a | b | c | d | ROR | $ROR_{025}$ | PRR | $\chi^2$ | IC | $IC_{025}$ | $IC_{975}$ |
|---|---|---|---|---|---|---|---|---|---|---|---|
| **Removing cases associated with gastrointestinal AEs** | | | | | | | | | | | |
| Leuprolide | 4286 | 155264 | 924087 | 35301271 | 1.05 | **1.02** | 1.05 | **11.71** | 0.07 | **0.02** | 0.11 |
| Triptorelin | 395 | 9468 | 927978 | 35447067 | 1.59 | **1.44** | 1.57 | **83.81** | 0.65 | **0.48** | 0.77 |
| Goserelin | 252 | 17265 | 928121 | 35439270 | 0.56 | 0.49 | 0.56 | 87.30 | -0.83 | -1.03 | -0.67 |
| Histrelin | 61 | 2025 | 928312 | 35454510 | 1.15 | 0.89 | 1.15 | 1.17 | 0.19 | -0.23 | 0.50 |
| Nafarelin | 18 | 574 | 928355 | 35455961 | 1.20 | 0.75 | 1.19 | 0.57 | 0.25 | -0.55 | 0.80 |
| **Prostate cancer** | | | | | | | | | | | |
| Leuprolide | 2281 | 117284 | 3189 | 290786 | 1.77 | **1.68** | 1.76 | **441.03** | 0.53 | **0.46** | 0.58 |
| Triptorelin | 35 | 3799 | 5435 | 404271 | 0.69 | 0.49 | 0.69 | 4.98 | -0.53 | -1.09 | -0.13 |
| Goserelin | 106 | 8598 | 5364 | 399472 | 0.92 | 0.76 | 0.92 | 0.75 | -0.12 | -0.44 | 0.11 |
| Histrelin | 6 | 304 | 5464 | 407766 | 1.47 | 0.66 | 1.46 | 0.89 | 0.50 | -0.92 | 1.41 |
| Nafarelin | 0 | 2 | 5470 | 408068 | 0.00 | --* | 0.00 | 0.03 | -0.07 | -10.40 | 1.91 |
| **Endometriosis** | | | | | | | | | | | |
| Leuprolide | 1670 | 29338 | 985 | 19795 | 1.14 | 1.06 | 1.14 | 10.66 | 0.07 | -0.01 | 0.13 |
| Triptorelin | 9 | 258 | 2646 | 48875 | 0.64 | 0.33 | 0.66 | 1.70 | -0.58 | -1.72 | 0.18 |
| Goserelin | 29 | 743 | 2626 | 48390 | 0.72 | 0.50 | 0.73 | 3.02 | -0.44 | -1.06 | 0.00 |
| Nafarelin | 11 | 258 | 2644 | 48875 | 0.79 | 0.43 | 0.80 | 0.60 | -0.31 | -1.34 | 0.38 |
| **Uterine leiomyoma** | | | | | | | | | | | |
| Leuprolide | 317 | 7990 | 225 | 6888 | 1.21 | 1.02 | 1.21 | 4.82 | 0.12 | -0.07 | 0.25 |
| Goserelin | 20 | 358 | 522 | 14520 | 1.55 | 0.98 | 1.52 | 3.60 | 0.57 | -0.18 | 1.10 |
| Nafarelin | 2 | 42 | 540 | 14836 | 1.31 | 0.32 | 1.29 | 0.14 | 0.29 | -2.30 | 1.68 |
| **Precocious puberty** | | | | | | | | | | | |

| | | | | | | | | | | | |
|---|---|---|---|---|---|---|---|---|---|---|---|
| Leuprolide | 207 | 4474 | 78 | 2419 | 1.43 | 1.10 | 1.42 | 7.20 | 0.16 | -0.07 | 0.32 |
| Triptorelin | 22 | 654 | 263 | 6239 | 0.80 | 0.51 | 0.80 | 1.00 | -0.28 | -1.00 | 0.22 |
| Histrelin | 42 | 1084 | 243 | 5809 | 0.93 | 0.66 | 0.93 | 0.20 | -0.09 | -0.60 | 0.28 |
| **Breast cancer** | | | | | | | | | | | |
| Leuprolide | 102 | 3976 | 14361 | 934141 | 1.67 | **1.37** | 1.65 | **26.46** | 0.72 | **0.39** | 0.95 |
| Triptorelin | 40 | 782 | 14423 | 937335 | 3.32 | **2.42** | 3.21 | **61.67** | 1.64 | **1.12** | 2.02 |
| Goserelin | 127 | 7363 | 14336 | 930754 | 1.12 | 0.94 | 1.12 | 1.59 | 0.16 | -0.13 | 0.37 |

Note: --*not calculable

Abbreviations: *DE*, depression; *IC*, information component; $IC_{025}$, lower side of the 95% confidence interval for IC; $IC_{975}$, upper side of the 95% confidence interval for IC; *PRR*, Proportional Reporting Ratio; *ROR*, Reporting Odds Ratio; $ROR_{025}$, lower side of the 95% confidence interval for ROR; $\chi^2$, chi-squared

A significant signal is present when $ROR_{025} > 1$, $IC_{025} > 0$ and $\chi^2 > 4$

**Table S8** Raw data and calculations for sensitivity analyses of SSI

| Drug | a | b | c | d | ROR | $ROR_{025}$ | PRR | $\chi^2$ | IC | $IC_{025}$ | $IC_{975}$ |
|---|---|---|---|---|---|---|---|---|---|---|---|
| **Removing cases associated with gastrointestinal AEs** | | | | | | | | | | | |
| Leuprolide | 284 | 159266 | 260386 | 35964972 | 0.25 | 0.22 | 0.25 | 653.14 | -2.01 | -2.20 | -1.86 |
| Triptorelin | 60 | 9803 | 260610 | 36114455 | 0.85 | 0.66 | 0.85 | 1.62 | -0.23 | -0.66 | 0.07 |
| Goserelin | 62 | 17455 | 260608 | 36106783 | 0.49 | 0.38 | 0.49 | 32.37 | -1.01 | -1.43 | -0.71 |
| Histrelin | 6 | 2080 | 260664 | 36122158 | 0.40 | 0.18 | 0.40 | 5.39 | -1.25 | -2.66 | -0.34 |
| Nafarelin | 4 | 588 | 260666 | 36123650 | 0.94 | 0.35 | 0.94 | 0.01 | -0.08 | -1.84 | 1.00 |
| **Prostate cancer** | | | | | | | | | | | |
| Leuprolide | 85 | 119480 | 166 | 293809 | 1.26 | 0.97 | 1.26 | 3.00 | 0.23 | -0.13 | 0.49 |
| Triptorelin | 12 | 3822 | 239 | 409467 | 5.38 | **3.01** | 5.37 | **40.61** | 2.14 | **1.17** | 2.81 |
| Goserelin | 16 | 8688 | 235 | 404601 | 3.17 | **1.91** | 3.17 | **22.22** | 1.51 | **0.67** | 2.10 |
| Histrelin | 0 | 310 | 251 | 412979 | 0.00 | --* | 0.00 | 0.19 | -0.46 | -10.78 | 1.52 |
| Nafarelin | 0 | 2 | 251 | 413287 | 0.00 | --* | 0.00 | 0.00 | 0.00 | -10.33 | 1.98 |
| **Endometriosis** | | | | | | | | | | | |
| Leuprolide | 130 | 30878 | 222 | 20558 | 0.39 | 0.31 | 0.39 | 77.65 | -0.69 | -0.99 | -0.49 |
| Triptorelin | 1 | 266 | 351 | 51170 | 0.55 | 0.08 | 0.55 | 0.37 | -0.63 | -4.41 | 1.06 |
| Goserelin | 7 | 765 | 345 | 50671 | 1.34 | 0.63 | 1.34 | 0.60 | 0.38 | -0.92 | 1.24 |
| Nafarelin | 0 | 269 | 352 | 51167 | 0.00 | --* | 0.00 | 1.85 | -2.22 | -12.54 | -0.24 |
| **Uterine leiomyoma** | | | | | | | | | | | |
| Leuprolide | 15 | 8292 | 41 | 7072 | 0.31 | 0.17 | 0.31 | 16.59 | -0.98 | -1.86 | -0.38 |
| Goserelin | 10 | 368 | 46 | 14996 | 8.86 | **4.44** | 8.65 | **55.78** | 2.49 | **1.41** | 3.21 |
| Nafarelin | 0 | 44 | 56 | 15320 | 0.00 | --* | 0.00 | 0.16 | -0.40 | -10.72 | 1.58 |
| **Precocious puberty** | | | | | | | | | | | |

| | | | | | | | | | | | |
|---|---|---|---|---|---|---|---|---|---|---|---|
| Leuprolide | 25 | 4656 | 15 | 2482 | 0.89 | 0.47 | 0.89 | 0.13 | -0.06 | -0.73 | 0.41 |
| Triptorelin | 7 | 669 | 33 | 6469 | 2.05 | 0.90 | 2.04 | 3.08 | 0.81 | -0.49 | 1.67 |
| Histrelin | 5 | 1121 | 35 | 6017 | 0.77 | 0.30 | 0.77 | 0.31 | -0.30 | -1.86 | 0.68 |
| **Breast cancer** | | | | | | | | | | | |
| Leuprolide | 5 | 4073 | 564 | 947938 | 2.06 | 0.85 | 2.06 | 2.71 | 0.91 | -0.66 | 1.89 |
| Triptorelin | 15 | 807 | 554 | 951204 | 31.91 | **19.02** | 31.35 | **429.37** | 3.97 | **3.10** | 4.57 |
| Goserelin | 15 | 7475 | 554 | 944536 | 3.42 | **2.05** | 3.42 | **24.98** | 1.64 | **0.77** | 2.24 |

Note: --*not calculable

Abbreviations: *IC*, information component; $IC_{025}$, lower side of the 95% confidence interval for IC; $IC_{975}$, upper side of the 95% confidence interval for IC; *PRR*, Proportional Reporting Ratio; *ROR*, Reporting Odds Ratio; $ROR_{025}$, lower side of the 95% confidence interval for ROR; *SSI*, suicide/self-injury; $\chi^2$, chi-squared

A significant signal is present when $ROR_{025} > 1$, $IC_{025} > 0$ and $\chi^2 > 4$

**Table S9** Raw data and calculations for subgroup analyses of DE

| Drug | a | b | c | d | ROR | $ROR_{025}$ | PRR | $\chi^2$ | IC | $IC_{025}$ | $IC_{975}$ |
|---|---|---|---|---|---|---|---|---|---|---|---|
| **Female** | | | | | | | | | | | |
| Leuprolide | 2840 | 58655 | 635512 | 29020104 | 2.21 | **2.13** | 2.16 | **1788.83** | 1.10 | **1.04** | 1.15 |
| Triptorelin | 336 | 5832 | 638016 | 29072927 | 2.63 | **2.35** | 2.54 | **319.50** | 1.34 | **1.16** | 1.47 |
| Goserelin | 170 | 9079 | 638182 | 29069680 | 0.85 | 0.73 | 0.86 | 4.23 | -0.22 | -0.48 | -0.04 |
| Histrelin | 51 | 1270 | 638301 | 29077489 | 1.83 | **1.38** | 1.80 | **18.43** | 0.83 | **0.37** | 1.17 |
| Nafarelin | 32 | 868 | 638320 | 29077891 | 1.68 | **1.18** | 1.66 | **8.48** | 0.71 | **0.12** | 1.13 |
| **Male** | | | | | | | | | | | |
| Leuprolide | 2627 | 128518 | 454741 | 17323480 | 0.78 | 0.75 | 0.78 | 160.98 | -0.35 | -0.41 | -0.30 |
| Triptorelin | 116 | 5413 | 457252 | 17446585 | 0.82 | 0.68 | 0.82 | 4.62 | -0.28 | -0.59 | -0.06 |
| Goserelin | 138 | 9097 | 457230 | 17442901 | 0.58 | 0.49 | 0.59 | 41.68 | -0.77 | -1.05 | -0.57 |
| Histrelin | 14 | 754 | 457354 | 17451244 | 0.71 | 0.42 | 0.71 | 1.65 | -0.47 | -1.38 | 0.15 |
| Nafarelin | 0 | 7 | 457368 | 17451991 | 0.00 | --* | 0.00 | 0.18 | -0.44 | -10.76 | 1.54 |
| **<18 years old in children** | | | | | | | | | | | |
| Leuprolide | 352 | 5428 | 41801 | 1895921 | 2.94 | **2.64** | 2.82 | **420.05** | 1.49 | **1.31** | 1.61 |
| Triptorelin | 110 | 1856 | 42043 | 1899493 | 2.68 | **2.21** | 2.58 | **108.87** | 1.36 | **1.04** | 1.58 |
| Goserelin | 0 | 38 | 42153 | 1901311 | 0.00 | --* | 0.00 | 0.84 | -1.41 | -11.73 | 0.57 |
| Histrelin | 31 | 1050 | 42122 | 1900299 | 1.33 | 0.93 | 1.32 | 2.49 | 0.40 | -0.20 | 0.82 |
| Nafarelin | 0 | 3 | 42153 | 1901346 | 0.00 | --* | 0.00 | 0.07 | -0.18 | -10.50 | 1.80 |
| **18–65 years old in adults** | | | | | | | | | | | |
| Leuprolide | 1855 | 48438 | 470930 | 20495207 | 1.76 | **1.59** | 1.64 | **474.59** | 0.71 | **0.64** | 0.77 |
| Triptorelin | 53 | 2725 | 472732 | 20540920 | 0.85 | 0.64 | 0.85 | 1.48 | -0.24 | -0.69 | 0.09 |
| Goserelin | 186 | 8437 | 472599 | 20535208 | 0.96 | 0.83 | 0.96 | 0.34 | -0.06 | -0.30 | 0.12 |

| | | | | | | | | | | | |
|---|---|---|---|---|---|---|---|---|---|---|---|
| Histrelin | 0 | 26 | 472785 | 20543619 | 0.00 | --* | 0.00 | 0.60 | -1.12 | -11.44 | 0.86 |
| Nafarelin | 29 | 712 | 472756 | 20542933 | 1.77 | **1.22** | 1.74 | **9.33** | 0.78 | **0.16** | 1.22 |
| **>65 years old in elders** | | | | | | | | | | | |
| Leuprolide | 1225 | 66646 | 128020 | 10650891 | 1.53 | **1.44** | 1.52 | **218.23** | 0.60 | **0.50** | 0.67 |
| Triptorelin | 20 | 2941 | 129225 | 10714596 | 0.56 | 0.36 | 0.57 | 6.70 | -0.80 | -1.55 | -0.28 |
| Goserelin | 82 | 6254 | 129163 | 10711283 | 1.09 | 0.87 | 1.09 | 0.57 | 0.12 | -0.25 | 0.38 |
| Histrelin | 4 | 177 | 129241 | 10717360 | 1.87 | 0.70 | 1.85 | 1.59 | 0.76 | -1.00 | 1.84 |
| Nafarelin | 0 | 3 | 129245 | 10717534 | 0.00 | --* | 0.00 | 0.04 | -0.10 | -10.42 | 1.88 |

Note: --*not calculable

Abbreviations: *DE*, depression; *IC*, information component; $IC_{025}$, lower side of the 95% confidence interval for IC; $IC_{975}$, upper side of the 95% confidence interval for IC; *PRR*, Proportional Reporting Ratio; *ROR*, Reporting Odds Ratio; $ROR_{025}$, lower side of the 95% confidence interval for ROR; $\chi^2$, chi-squared

A significant signal is present when $ROR_{025} > 1$, $IC_{025} > 0$ and $\chi^2 > 4$

**Table S10** Raw data and calculations for subgroup analyses of SSI

| Drug | a | b | c | d | ROR | $ROR_{025}$ | PRR | $\chi^2$ | IC | $IC_{025}$ | $IC_{975}$ |
|---|---|---|---|---|---|---|---|---|---|---|---|
| **Female** | | | | | | | | | | | |
| Leuprolide | 267 | 61228 | 159581 | 29496035 | 0.81 | 0.71 | 0.81 | 12.39 | -0.31 | -0.51 | -0.16 |
| Triptorelin | 40 | 6128 | 159808 | 29551135 | 1.21 | 0.88 | 1.21 | 1.41 | 0.27 | -0.26 | 0.64 |
| Goserelin | 34 | 9215 | 159814 | 29548048 | 0.68 | 0.49 | 0.68 | 5.01 | -0.54 | -1.11 | -0.14 |
| Histrelin | 4 | 1317 | 159844 | 29555946 | 0.56 | 0.21 | 0.56 | 1.36 | -0.76 | -2.52 | 0.32 |
| Nafarelin | 4 | 896 | 159844 | 29556367 | 0.83 | 0.31 | 0.83 | 0.15 | -0.25 | -2.01 | 0.83 |
| **Male** | | | | | | | | | | | |
| Leuprolide | 106 | 131039 | 109296 | 17668925 | 0.13 | 0.11 | 0.13 | 611.33 | -2.91 | -3.23 | -2.68 |
| Triptorelin | 19 | 5510 | 109383 | 17794454 | 0.56 | 0.36 | 0.56 | 6.50 | -0.81 | -1.58 | -0.28 |
| Goserelin | 19 | 9216 | 109383 | 17790748 | 0.34 | 0.21 | 0.34 | 24.98 | -1.55 | -2.32 | -1.01 |
| Histrelin | 2 | 766 | 109400 | 17799198 | 0.42 | 0.11 | 0.43 | 1.55 | -1.05 | -3.65 | 0.34 |
| Nafarelin | 0 | 7 | 109402 | 17799957 | 0.00 | --* | 0.00 | 0.04 | -0.12 | -10.44 | 1.86 |
| **<18 years old in children** | | | | | | | | | | | |
| Leuprolide | 31 | 5749 | 24012 | 1913710 | 0.43 | 0.30 | 0.43 | 23.30 | -1.19 | -1.79 | -0.77 |
| Triptorelin | 9 | 1957 | 24034 | 1917502 | 0.37 | 0.19 | 0.37 | 9.78 | -1.39 | -2.52 | -0.62 |
| Goserelin | 0 | 38 | 24043 | 1919421 | 0.00 | --* | 0.00 | 0.48 | -0.96 | -11.28 | 1.02 |
| Histrelin | 5 | 1076 | 24038 | 1918383 | 0.37 | 0.15 | 0.37 | 5.31 | -1.33 | -2.90 | -0.35 |
| Nafarelin | 0 | 3 | 24043 | 1919456 | 0.00 | --* | 0.00 | 0.04 | -0.10 | -10.43 | 1.88 |
| **18–65 years old in adults** | | | | | | | | | | | |
| Leuprolide | 174 | 50119 | 175093 | 20791044 | 0.41 | 0.36 | 0.41 | 145.16 | -1.27 | -1.52 | -1.09 |
| Triptorelin | 15 | 2763 | 175252 | 20838400 | 0.65 | 0.39 | 0.65 | 2.90 | -0.61 | -1.48 | -0.01 |
| Goserelin | 38 | 8585 | 175229 | 20832578 | 0.53 | 0.38 | 0.53 | 16.13 | -0.91 | -1.45 | -0.53 |

| | | | | | | | | | | | |
|---|---|---|---|---|---|---|---|---|---|---|---|
| Histrelin | 0 | 26 | 175267 | 20841137 | 0.00 | --* | 0.00 | 0.22 | -0.52 | -10.84 | 1.46 |
| Nafarelin | 3 | 738 | 175264 | 20840425 | 0.48 | 0.16 | 0.49 | 1.65 | -0.93 | -3.00 | 0.27 |
| **>65 years old in elders** | | | | | | | | | | | |
| Leuprolide | 41 | 67830 | 23562 | 10755349 | 0.28 | 0.20 | 0.28 | 77.73 | -1.84 | -2.36 | -1.47 |
| Triptorelin | 8 | 2953 | 23595 | 10820226 | 1.24 | 0.62 | 1.24 | 0.38 | 0.29 | -0.92 | 1.09 |
| Goserelin | 5 | 6331 | 23598 | 10816848 | 0.36 | 0.15 | 0.36 | 5.62 | -1.38 | -2.94 | -0.39 |
| Histrelin | 0 | 181 | 23603 | 10822998 | 0.00 | --* | 0.00 | 0.39 | -0.84 | -11.16 | 1.14 |
| Nafarelin | 0 | 3 | 23603 | 10823176 | 0.00 | --* | 0.00 | 0.01 | -0.02 | -10.34 | 1.96 |

Note: --*not calculable

Abbreviations: *IC*, information component; $IC_{025}$, lower side of the 95% confidence interval for IC; $IC_{975}$, upper side of the 95% confidence interval for IC; *PRR*, Proportional Reporting Ratio; *ROR*, Reporting Odds Ratio; $ROR_{025}$, lower side of the 95% confidence interval for ROR; *SSI*, suicide/self-injury; $\chi^2$, chi-squared

A significant signal is present when $ROR_{025} > 1$, $IC_{025} > 0$ and $\chi^2 > 4$

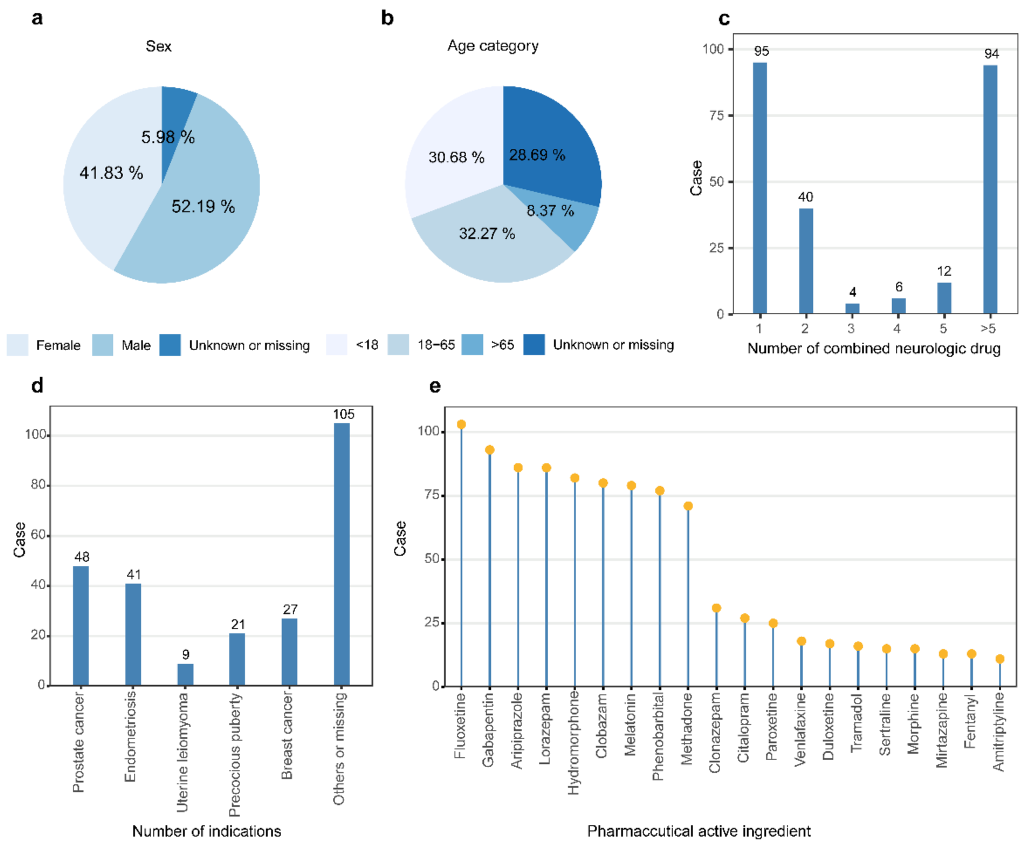


**Fig. S1** Clinical data of 251 patients who developed DE while being treated with a combination of GnRH-a and neurologic drugs. (**a**) Sex distribution; (**b**) Age distribution; (**c**) Number of combined neurological drugs; (**d**) Number of indications; (**e**) Combined neurological drugs involved and their distribution. *DE*, depression

**Table S11** $IC_{025}$ and $\Omega_{025}$ of the top 20 neurological drugs co-administered with GnRH-a in patients occurring DE

| Drug1 | Drug2 | N | $n_{111}$ | $n_{110}$ | $n_{101}$ | $n_{100}$ | $n_{011}$ | $n_{010}$ | $n_{001}$ | $n_{000}$ | IC($IC_{025}$–$IC_{975}$) | Ω($\Omega_{025}$–$\Omega_{975}$) |
|---|---|---|---|---|---|---|---|---|---|---|---|---|
| GnRH-a | Fluoxetine | 103 | 208 | 783 | 6384 | 238951 | 8649 | 164649 | 1176111 | 50930324 | 1.14 (1.10–1.18) | 1.97 (1.77–2.17) |
| GnRH-a | Gabapentin | 93 | 186 | 939 | 6406 | 238795 | 13956 | 339259 | 1170804 | 50755714 | 0.88 (0.84–0.90) | 1.93 (1.73–2.14) |
| GnRH-a | Aripiprazole | 86 | 188 | 689 | 6404 | 239045 | 12064 | 277457 | 1172696 | 50817516 | 0.86 (0.83–0.89) | 2.24 (2.03–2.44) |
| GnRH-a | Lorazepam | 86 | 180 | 632 | 6412 | 239102 | 10314 | 160895 | 1174446 | 50934078 | 1.64 (1.59–1.68) | 1.79 (1.58–2.00) |
| GnRH-a | Hydromorphone | 82 | 177 | 813 | 6415 | 238921 | 68990 | 247965 | 1115770 | 50847008 | 3.24 (3.21–3.26) | -0.30 (-0.52– (-0.09)) |
| GnRH-a | Clobazam | 80 | 172 | 536 | 6420 | 239198 | 1130 | 33945 | 1183630 | 51061028 | 0.73 (0.58–0.84) | 2.75 (2.53–2.96) |
| GnRH-a | Melatonin | 79 | 170 | 567 | 6422 | 239167 | 496 | 19218 | 1184264 | 51075755 | 0.70 (-0.24–1.34) | 2.98 (2.76–3.20) |
| GnRH-a | Phenobarbital | 77 | 169 | 534 | 6423 | 239200 | 560 | 28108 | 1184200 | 51066865 | -1.19 (-1.88– (-0.70)) | 3.17 (2.95–3.38) |
| GnRH-a | Methadone | 71 | 154 | 519 | 6438 | 239215 | 15692 | 95055 | 1169068 | 50999918 | 1.00 (0.91–1.06) | 0.66 (0.43–0.89) |
| GnRH-a | Clonazepam | 31 | 56 | 445 | 6536 | 239289 | 12434 | 194179 | 1172326 | 50900794 | 1.41 (1.36–1.44) | 0.80 (0.42–1.17) |
| GnRH-a | Citalopram | 27 | 55 | 549 | 6537 | 239185 | 15632 | 319415 | 1169128 | 50775558 | 1.11 (1.07–1.13) | 0.84 (0.46–1.22) |
| GnRH-a | Paroxetine | 25 | 50 | 405 | 6542 | 239329 | 13974 | 219893 | 1170786 | 50875080 | 1.44 (1.40–1.46) | 0.78 (0.38–1.18) |
| GnRH-a | Venlafaxine | 18 | 40 | 582 | 6552 | 239152 | 19432 | 318447 | 1165328 | 50776526 | 1.48 (1.45–1.50) | 0.06 (-0.38–0.51) |
| GnRH-a | Duloxetine | 17 | 28 | 224 | 6564 | 239510 | 22325 | 329392 | 1162435 | 50765581 | 1.68 (1.65–1.69) | 0.70 (0.17–1.24) |
| GnRH-a | Tramadol | 16 | 16 | 365 | 6576 | 239369 | 41815 | 323190 | 1142945 | 50771783 | 1.65 (1.62–1.68) | -1.47 (-2.17– (-0.76)) |
| GnRH-a | Sertraline | 15 | 30 | 246 | 6562 | 239488 | 15276 | 274787 | 1169484 | 50820186 | 1.22 (1.19–1.25) | 0.92 (0.40–1.44) |
| GnRH-a | Morphine | 15 | 15 | 567 | 6577 | 239167 | 76347 | 397531 | 1108413 | 50697442 | 1.99 (1.96–2.01) | -2.64 (-3.37– (-1.91)) |
| GnRH-a | Mirtazapine | 13 | 31 | 208 | 6561 | 239526 | 6431 | 133043 | 1178329 | 50961930 | 0.99 (0.93–1.04) | 1.34 (0.83–1.85) |
| GnRH-a | Fentanyl | 13 | 13 | 514 | 6579 | 239220 | 30812 | 363871 | 1153948 | 50731102 | 1.35 (1.32–1.37) | -1.70 (-2.48– (-0.91)) |
| GnRH-a | Amitriptyline | 11 | 20 | 211 | 6572 | 239523 | 4286 | 103277 | 1180474 | 50991696 | 0.66 (0.53–0.76) | 0.95 (0.32–1.58) |

Note: A drug-drug interaction signal was considered significant when $\Omega_{025} > 0$; a significant signal between neurological drug and DE was observed when $IC_{025} > 0$

Abbreviations: *DE*, depression; *IC*, information component; $IC_{025}$, lower side of the 95% confidence interval for IC; $IC_{975}$, upper side of the 95% confidence interval for IC; *N*, number of patients reported; *n*, number of adverse drug reactions reported; $\Omega_{025}$, lower side of the 95% confidence interval for Ω; $\Omega_{975}$, upper side of the 95% confidence interval for Ω

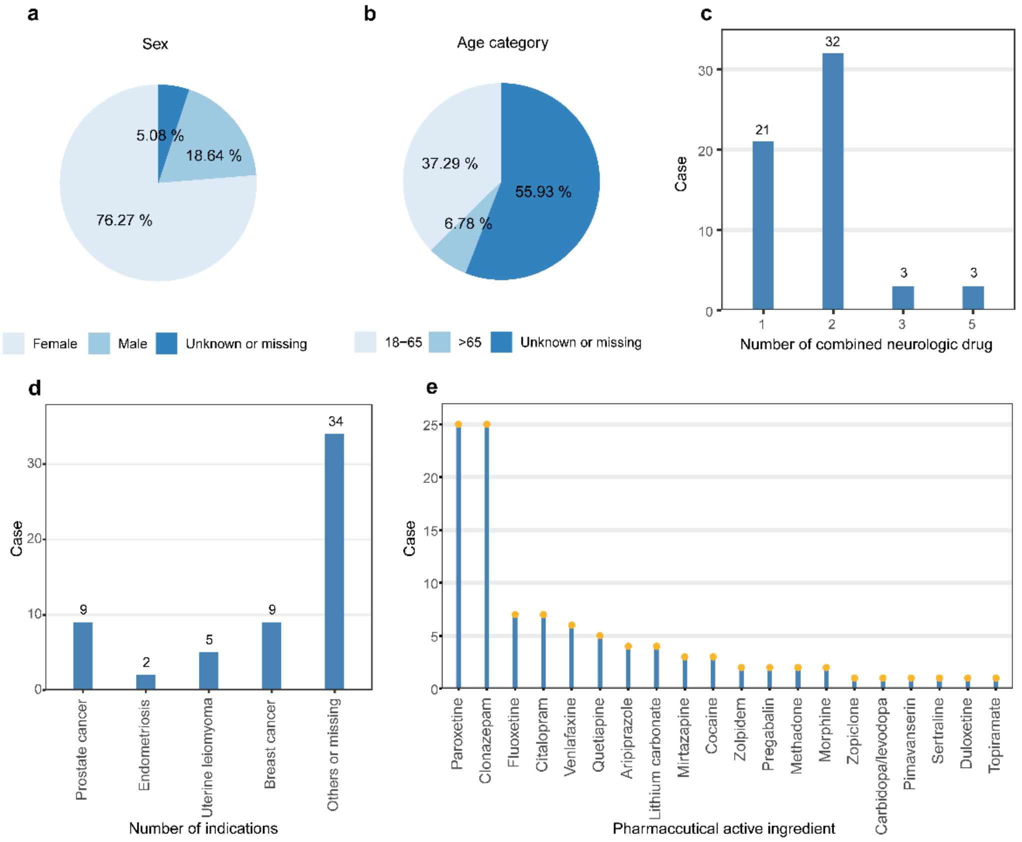


**Fig. S2** Clinical data of 59 patients who developed SSI while being treated with a combination of GnRH-a and neurologic drugs. (**a**) Sex distribution; (**b**) Age distribution; (**c**) Number of combined neurological drugs; (**d**) Number of indications; (**e**) Combined neurological drugs involved and their distribution. *SSI*, suicide/self-injury

**Table S12** $IC_{025}$ and $\Omega_{025}$ of the top 20 neurological drugs co-administered with GnRH-a in patients occurring SSI

| Drug1 | Drug2 | N | $n_{111}$ | $n_{110}$ | $n_{101}$ | $n_{100}$ | $n_{011}$ | $n_{010}$ | $n_{001}$ | $n_{000}$ | IC($IC_{025}$–$IC_{975}$) | Ω($\Omega_{025}$–$\Omega_{975}$) |
|---|---|---|---|---|---|---|---|---|---|---|---|---|
| GnRH-a | Paroxetine | 25 | 25 | 430 | 510 | 245361 | 9167 | 224700 | 287636 | 51758230 | 2.66 (2.62–2.69) | 0.48 (-0.09–1.04) |
| GnRH-a | Clonazepam | 25 | 25 | 476 | 510 | 245315 | 10013 | 196600 | 286790 | 51786330 | 2.59 (2.53–2.63) | 0.04 (-0.52–0.61) |
| GnRH-a | Fluoxetine | 7 | 8 | 983 | 527 | 244808 | 10140 | 163158 | 286663 | 51819772 | 3.15 (3.10–3.18) | -2.78 (-3.78– (-1.78)) |
| GnRH-a | Citalopram | 7 | 8 | 596 | 527 | 245195 | 16528 | 318519 | 280275 | 51664411 | 2.96 (2.93–2.99) | -1.81 (-2.81– (-0.81)) |
| GnRH-a | Venlafaxine | 6 | 6 | 616 | 529 | 245175 | 14216 | 323663 | 282587 | 51659267 | 2.65 (2.61–2.68) | -2.04 (-3.19– (-0.88)) |
| GnRH-a | Quetiapine | 5 | 5 | 174 | 530 | 245617 | 21160 | 453790 | 275643 | 51529140 | 2.74 (2.71–2.77) | -0.62 (-1.89–0.64) |
| GnRH-a | Aripiprazole | 4 | 4 | 873 | 531 | 244918 | 8954 | 280567 | 287849 | 51702363 | 2.26 (2.22–2.29) | -2.62 (-4.03– (-1.20)) |
| GnRH-a | Lithium carbonate | 4 | 4 | 81 | 531 | 245710 | 2189 | 64579 | 294614 | 51918351 | 1.76 (1.60–1.88) | 0.45 (-0.96–1.87) |
| GnRH-a | Mirtazapine | 3 | 3 | 236 | 532 | 245555 | 7128 | 132346 | 289675 | 51850584 | 2.96 (2.90–3.00) | -2.13 (-3.76– (-0.49)) |
| GnRH-a | Cocaine | 3 | 3 | 18 | 532 | 245773 | 2445 | 42039 | 294358 | 51940891 | 4.48 (4.23–4.67) | 1.08 (-0.55–2.71) |
| GnRH-a | Zolpidem | 2 | 3 | 124 | 532 | 245667 | 9374 | 120829 | 287429 | 51862101 | 3.28 (3.23–3.32) | -1.46 (-3.09–0.17) |
| GnRH-a | Pregabalin | 2 | 2 | 163 | 533 | 245628 | 6644 | 554811 | 290159 | 51428119 | 0.74 (0.68–0.78) | 0.03 (-1.97–2.03) |
| GnRH-a | Methadone | 2 | 2 | 671 | 533 | 245120 | 3273 | 107474 | 293530 | 51875456 | 2.63 (2.53–2.70) | -3.03 (-5.03– (-1.03)) |
| GnRH-a | Morphine | 2 | 2 | 580 | 533 | 245211 | 5308 | 468570 | 291495 | 51514360 | 1.58 (1.51–1.63) | -1.49 (-3.49–0.51) |
| GnRH-a | Zopiclone | 1 | 2 | 85 | 533 | 245706 | 4479 | 92028 | 292324 | 51890902 | 1.24 (1.06–1.38) | -0.86 (-2.86–1.14) |
| GnRH-a | Carbidopa/levodopa | 1 | 1 | 51 | 534 | 245740 | 547 | 235477 | 296256 | 51747453 | -1.45 (-1.61– (-1.33)) | 0.91 (-1.91–3.74) |
| GnRH-a | Pimavanserin | 1 | 1 | 30 | 534 | 245761 | 131 | 101957 | 296672 | 51880973 | -2.15 (-2.44– (-1.94)) | 1.15 (-1.68–3.98) |
| GnRH-a | Sertraline | 1 | 1 | 275 | 534 | 245516 | 12538 | 277525 | 284265 | 51705405 | 2.76 (2.73–2.79) | -3.05 (-5.88– (-0.22)) |
| GnRH-a | Duloxetine | 1 | 1 | 251 | 534 | 245540 | 10285 | 341432 | 286518 | 51641498 | 2.20 (2.16–2.23) | -2.39 (-5.22–0.44) |
| GnRH-a | Topiramate | 1 | 1 | 59 | 534 | 245732 | 3415 | 138043 | 293388 | 51844887 | 1.86 (1.78–1.92) | -0.38 (-3.21–2.45) |

Note: A drug-drug interaction signal was considered significant when $\Omega_{025} > 0$; a significant signal between neurological drug and SSI was observed when $IC_{025} > 0$

Abbreviations: *IC*, information component; $IC_{025}$, lower side of the 95% confidence interval for IC; $IC_{975}$, upper side of the 95% confidence interval for IC; *N*, number of patients reported; *n*, number of adverse drug reactions reported; *SSI*, suicide/self-injury; $\Omega_{025}$, lower side of the 95% confidence interval for Ω; $\Omega_{975}$, upper side of the 95% confidence interval for Ω
